# Surgery

**Published:** 1878-06

**Authors:** 


### SURGERY.

Aphorisms in Fractures.—Professor R. C. Cowling, in a paper read before the Central Kentucky Medical Society, in July, 1877, gives the following as aphorisms concerning fractures which every surgeon will admit are of great practical value:

1. In establishing the diagnosis of fracture, crepitus is the most satisfactory sign; nevertheless it is not always necessary or desirable to obtain it.

2. There is frequently a physiognomy about fracture which declares the nature of the lesion; notably in Colles's fracture of the radius, ordinary fractures of clavicle and neck of femur.

3. The sensation of crepitus in recent fracture is *sui generis* and unmistakable. When once detected, further attempts to elicit should not be made, except for the benefit of an accompanying physician who may be in doubt as to the nature of the injury.

4. Crepitus can not be had in impacted fracture except to the injury of the patient. It is difficult to obtain it in intra-capsular fracture of the femoral neck under all circumstances.

5. "Loss of function" as a symptom is all important in establishing diagnosis in certain fractures.

6. When any doubt exists as to diagnosis in the examination it should be conducted under an anæsthetic.

7. The chief difficulty in the differentiation of fracture is from sprain.

8. The prognosis in simple fracture is always favorable when injury is confined to shaft of bone, even if there be comminution.

9. In multiple fracture, when simple, the healing process is not retarded.

10. In fracture involving a joint, more or less stiffness is to be feared.

11. With the improved methods of treatment, the danger to life and limb in compound fracture has been reduced to such an extent, that former laws for determining the question of amputation are to be recast.

12. The best time to dress any fracture is immediately after its occurrence.

13. Temporary dressings are to be used only when permanent dressings are not to be had, or to move patient.

14. Under proper treatment immediately instituted, swelling will probably be prevented.

15. The indications for treatment are, first, reduction of fragments; second, their immobilization.

16. The dressing of the fractures is best done under an anæsthetic, to secure comfort to patient and prevent muscular spasm.

17. Perfect immobilization is only obtained when joints contiguous to the fracture are secured, and there is no law more important than this in fractures of the lower extremity.

18. One of the commonest reasons for failure and disaster in the treatment of fracture arises from the fact that bone and muscle only are considered, blood-vessels and nerves are left out of sight.

19. Carved and manufactured splints generally fit nobody, and are to be rejected as not only expensive, but damaging. Dealboard, pasteboard, and materials for the plastic apparatus are all that is needed.

20. The application of a bandage immediately to the skin has resulted in such disaster that it is one of the curiosities of surgery how it could be repeated at this day.

21. Evenness of pressure is only to be obtained by the proper lining of the splints with cotton, or in compound fracture, with oakum.

22. The cotton to be used is preferably that known as "batting." Only the best should be used. The layers should be unbroken, It should be used freely, especially over bony prominences.

23. Continued extension and counter-extension are, as a rule, not necessary to prevent shortening. This is best done by removing the causes which lead to muscular spasm; first, by early interference; second, by as complete reposition of the fragments as possible; third, by smooth application of batting to the limb; fourth, by the equal pressure of the bandage, going from the distal end of limb to a point beyond the joint above the fracture; fifth, by accurate filling of splints or plaster; sixth, by as little interference afterward as possible.

24. Angular deformity is best overcome by the same measures as are used in longitudinal deformity. Compresses are to be avoided.

25. Bandages are made of cotton or cheap flannel, with selvage edge torn off, and thoroughly shrunk.

26. Comfort is the sign that a fracture has been properly dressed.

27. Frequent dressing for purposes of examination is useless and hurtful.

28. Whenever possible, after dressing a fracture, it should be seen again in a few hours, and daily in its earlier stages.

29. If in a consultation, a difference of opinion in regard to treatment arises, one should yield. Compromises often result badly, while fractures may do well under almost any reputable method properly pursued.—*Louisville Med. Times.*

